# Landslide susceptibility assessment via the information value-coupled machine learning models

**DOI:** 10.1371/journal.pone.0333055

**Published:** 2025-10-21

**Authors:** Yamei Wang, Zizhao Zhang, Xikun Yu, Chenxin Liu

**Affiliations:** 1 School of Geology and Mining Engineering, Xinjiang University, Urumqi, China; 2 Research Base of Xinjiang University, State Key Laboratory of Deep Rock Mechanics and Underground Engineering, Urumqi, China; Guizhou University, CHINA

## Abstract

Collapses and landslides are frequent in the southern mountainous areas of the economic zone on the northern slopes of the Tianshan Mountains in Xinjiang, and an accurate assessment of susceptibility can effectively avoid potential risks, which is crucial for the prevention and control of geological hazards. To obtain precise and reliable references for the prevention of landslide, a total of 10 landslide conditioning factors (e.g., elevation, slope degree, slope aspect, curvature, relief, engineering geological lithology, landform types, land use, distance to rivers, as well as distance to roads) were selected for the multicollinearity analysis. The evaluation index system was established in the present research to assess the landslide susceptibility with the combination of traditional statistical methods and machine learning models. Both the information value-maximum entropy coupled model (I-MaxEnt) and the information value-logistic regression coupled model (I-LR) were proposed to assess landslide susceptibility in the Tianshan northern slope economic belt after the detailed evaluation on the information value model (I), logistic regression model (LR), and maximum entropy model (MaxEnt). Comparative discussions on the receiver operating characteristic (ROC)curves revealed that the area under the curve(AUC) values of the I-MaxEnt and I-LR coupled models were 0.907 and 0.941, respectively, indicating the superior accuracy of the I-LR model. Furthermore, the results obtained from the I-LR model were more consistent with the actual situation as verified by field validation. That is, the I-LR model is much more suitable in assessing the landslide susceptibility in the given research region attributed to its high accuracy and reliability.The results of this research provide a reliable basis for disaster prevention and mitigation in this study area.

## 1. Introduction

The landslide attributed to the combined influence of internal and external geological and geomorphological processes is one of the most serious geological hazards threatening to local resident lives and economic development in mountain regions [1–3]. Attributed to its vast territory, complex geological and climatic conditions, intense tectonic movement, seismic activity and human activities, China is one of the countries with prominent geological disaster issues around the world [4,5]. The economic zone on the northern slopes of the Tianshan Mountains in central Xinjiang is the most densely populated and productive urban area, and the southern mountainous areas are of high value for development, but geological hazards are frequent in the region, and existing studies have not evaluated specific susceptibility for this region.As historically statistic, there are about 1530 geological disasters occurred within the southern mountainous of the Economic Belt on the Northern Slope of the Tianshan Mountains, including 836 collapses and 331 landslides. these geological disasters have significantly restricted the development of local tourism, mining activities, and transportation industry. It is thus of great significance to conduct the landslide susceptibility assessment for regional disaster prevention and reduction, which can also provide reference to the scientific planning and management.

Currently, the landslide susceptibility assessment methods can be classified into two groups, one is the qualitative method and the other is the quantitative method [6], either of which has its own advantages and disadvantages. Because that the natural environmental conditions (e.g., geological conditions and climate environment) and triggering factors for the geological disaster are different from each other at different regions, it is not easy to get the standard susceptibility assessment results. It is therefore requested to establish unique slope susceptibility assessment models for different research area. Since statistical analysis methods cannot explain the nonlinear relationship between various types of disaster-causing factors and the evaluation accuracy is low [7], machine learning models have emerged. Machine learning has a strong adaptive learning ability, which can better capture the nonlinear relationship between factors and landslides [8]. However, a single evaluation model has certain limitations, and it is difficult to appropriately represent the input data of landslide susceptibility in line with the field conditions, resulting in the model not being able to accurately fit the optimal function and the true distribution of the sample set, and coupled modelling has been considered as an effective technique to solve this problem [9]. Therefore, combining statistical methods with machine learning models has become a research trend in recent years [10].

Based on the landslides in Jiuzhaigou scenic area after the earthquake in 2017, Luo et al. combined the coefficient of certainty (CF) and the entropy index (IOE) with the logistic regression (LR) model and the support vector machine (SVM), and selected nine indexes such as tectonic factor, topographic factor, geological factor, and other factors, to evaluate the susceptibility to landslides in Jiuzhaigou, and the results showed that the use of the coupled model The AUC value reaches 0.847, which is more reasonable and accurate than the single model evaluation results such as deterministic coefficient model and logistic regression model, and provides a basis for disaster prevention and mitigation in the scenic area [11]. Li et al. for Qinghai Shatangchuan basin loess Liangxuan landslide problem, the use of information quantity model, logistic regression model and the two coupled model for susceptibility assessment, this is because of the wide application of quantitative assessment methods, which information quantity and logistic regression model is commonly used but has limitations, so this paper carries out a comparative study and the establishment of the coupled model in order to improve the assessment method [12]. It is found that the coupled model has the coefficients of the regression analysis after the normalisation and covariance test of the informativeness values, and the fitted equations are superimposed on the layers to obtain the grading map, which is generally consistent with the actual distribution of landslides and has the highest success rate, and is more suitable for quantitative assessment of landslide susceptibility in this watershed. Chen et al. combined RF with SI, CF and IOE to assess landslide susceptibility in Taibai County [10]. The proposed new method combining RF with SI, CF and IOE is more effective than the integrated method based on Support Vector Machines (SVM), in which the RF-CF model has the best performance in terms of landslide and non-landslide image classification performance, accuracy and Kappa index, and also the highest Area Under the ROC Curve (AUC), which is the most effective model to help in landslide disaster prevention management and decision making. This model further highlights the conclusion that coupled models are better than single models and are useful for landslide disaster prevention management and decision making, and can also be used as a reference for other regional studies and geo-environmental problems.Jaafari applied frequency ratio and entropy index models for LSM and the results showed that both models were effective and easy to use in the area, with the entropy index model predicting better [13]. The mapping of landslide susceptibility can provide a basis for decision making for activities such as forest road construction and timber harvesting, which can help managers to reduce risks. Lee et al. used probabilistic analysis and logistic regression model to analyse the relationship between landslides and various factors and to draw a susceptibility map using Yongin City, South Korea as the study object [14]. The results of the study can be used as a reference for planners and engineers to assist in slope management and land use planning, but may have limited applicability to specific sites with large localised geological and geographic variations.Liang F et al. used the informativeness model (I), the coefficient of determination (CF) method and the I-CF coupled model to carry out a study on the evaluation of the susceptibility of landslides to geological hazards in Guiding County. The results show that the accuracy of the I-CF coupled model is better than that of the single evaluation model, and it is proved that the contribution rate of the fault factor is higher [15]. This is because faulting is an important phenomenon in geological formations, which usually marks the rupture and misalignment of rock and soil bodies. In the vicinity of faults, geological action tends to be more active, which significantly affects the stability of geotechnical bodies, thus increasing the risk of geological hazards [16]. Xin Wei et al. used LR and convolutional neural network (CNN) as the data-driven module, and infinite slope stability analysis model as the physical mechanics module, and established two new hybrid models, i.e., Hybrid Model I (LR + Infinite Slope Stability Analysis Model) and Hybrid Model II (CNN + Infinite Slope Stability Analysis Model) [17]. Among them, the physical mechanics module converts the stratigraphic lithology evaluation factors into safety coefficients with fixed value intervals and uses them as additional input evaluation factors for the data-driven module, which effectively solves the generalisation problem of the data-driven model due to the inter-area stratigraphic lithology differences and unlabelled conditions, and solves the problem of the incomplete landslide inventory due to the non-landslide samples by setting a threshold of safety coefficients for the screening of the significant prediction uncertainty. Li took Xishan Coalfield as the study area, coupled three machine learning models, namely logistic regression, random forest and support vector machine, respectively, using informative methods to evaluate landslide susceptibility in the study area, and selected the optimal model through the ROC curve [18]. The results show that the coupled models can effectively evaluate landslide hazard susceptibility, among which the I-LR model has the best partitioning effect and classification accuracy, which further verifies the conclusion that the effect of the coupled models is better than that of a single model, and provides ideas for improving the accuracy of landslide susceptibility evaluation.

The economic zone on the north slope of Tianshan Mountain has a pivotal influence on the whole Xinjiang, and its natural geography and geological environment have special background conditions, so it is of great significance to construct a collapse and landslide susceptibility assessment model suitable for this area. In this paper, the traditional statistical methods of information model (I) coupled with maximum entropy model (MaxEnt) and logistic regression model (LR) are selected to evaluate the susceptibility, analyse and compare the evaluation results, and the results of the research provide a reliable basis for disaster prevention and mitigation in the study area, and at the same time provide reference for the construction of the evaluation model of the susceptibility to geologic hazards in other areas.

## 2. Materials and methods

### 2.1. Overview

**Fig 1:**
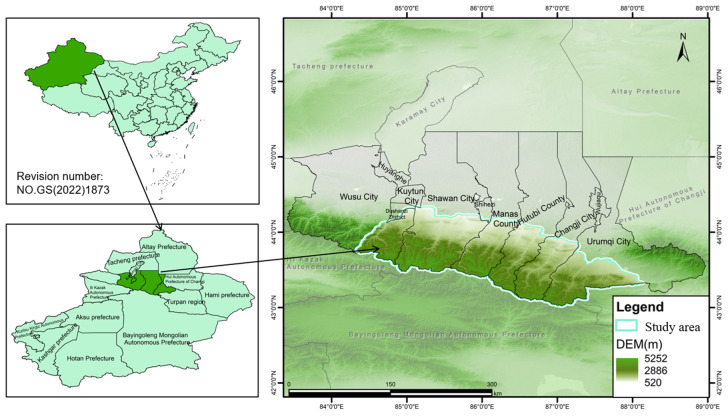
Location of the study area.

The southern mountainous region of the Economic Belt on the Northern Slope of the Tianshan Mountains in Xinjiang with an area of 21381.22 km^2^ extends from Urumqi to Wusu. As depicted in Fig 1, the geographical coordinates of the study area ranges from 43°00’N to 44°20’N and 84°25’E to 88°15’E, the terrain of which is higher in the south with a complex landscape including plains, hills, and mountains. Situated in the heartland of the Eurasian continent which is far from the ocean, it is of a typical temperate continental arid climate with hot-dry winds in the summer and cold winter invasions. Attributed to the significant topographical variations, the maximum elevation difference of which is over 4000m, there is a micro-climates from south to north along the vertical distribution. The plain area is generally featured with a mid-temperate continental arid climate with distinct four-seasons, while no distinct four-season climate can be seen from the mountainous area only with the cold climate. Notice that the southern part of the study area receives more precipitation and less sunshine, there is a shorter frost-free period, while the northern part receives less precipitation but more sunshine, with a longer frost-free period.

### 2.2. Data sources

This study is based on risk census data from prefecture-level cities. Through comprehensive field surveys, a database for evaluating the risk of landslides and rockfalls was constructed. During the survey, the team strictly followed geological disaster investigation standards, using high-precision differential GPS (positioning error < 1 m), 0.2 m resolution drone aerial surveying technology, and remote sensing interpretation to accurately locate disaster sites and calculate their scale. At least three high-definition images from different angles were retained for each site. During the data processing phase, historical remote sensing data, field records, and survey materials were cross-referenced to verify stable landslide bodies, identify new hazard points, and ensure the accuracy and validity of the data. Ultimately, 1,167 landslide and slope failure hazard sites were identified in the study area, including 836 landslide sites and 331 slope failure sites, providing reliable data support for subsequent hazard modeling. The non-landslide data are points selected using the ‘Create Random Points’ function of the ArcMAP 10.8 software within the non-vulnerable areas obtained from the information volume model.For the topographic and geomorphological types, based on the collection of risk survey and evaluation information of counties and cities conducted in the previous period, we compared the topographic and geomorphological types with those of the whole of Xinjiang, and verified and refined them based on satellite images.More information about the data sources, accuracy, and respective purposes are listed in Table 1 for ease of reference.

**Table 1:** Detailed information about the database.

Serial number	Data types	Source of data	Purpose of data
1	DEM	USGS National Map Viewer (https://www.usgs.gov/tools/national-map-viewer)	Extract slope, slope direction, curvature, undulation
2	Road data	Natural Earth	Extraction distance from road
3	Water system data	Natural Earth	Extraction distance from water system
4	Land-use data	Natural Earth	Extraction of land use types
5	Topographic and geomorphological data	Natural Earth	Extraction of topographic landform types
6	Engineering geological rock group data	Natural Earth	Extraction of engineering geological rock group types

### 2.3. Evaluation factors

Because the landslide susceptibility is mainly comprehensively affected by the internal geological factors and external environmental elements, these two aspects should be well considered during the selection of evaluation indicators [11]. Based on the comprehensive field investigation and analysis on previous researches, the evaluation unit with a 30m × 30m grids were determined in the present research and ten evaluation indicators were adopted for the landslide susceptibility assessment in this study region. These critical indicators include elevation, distance to rivers, the geomorphic type, slope angle, aspect of slope, topographical relief, curvature, distance to roads, landuse and the engineering geological rock group.

#### 2.3.1. Hydrological conditions.

These monitored slope hazards are mainly distributed in bands along both sides of the surface water system. Since water systems generally represent the scale of regional surface runoff and the density of river valleys, erosion is considered to be one of the important triggering factors for slope hazards [19]. The degree of development and distribution density of water systems are generally positively correlated with the surface erosion capacity, therefore, widely distributed water systems are more likely to trigger geological disasters due to their stronger surface erosion capacity [20].

**Fig 2:**
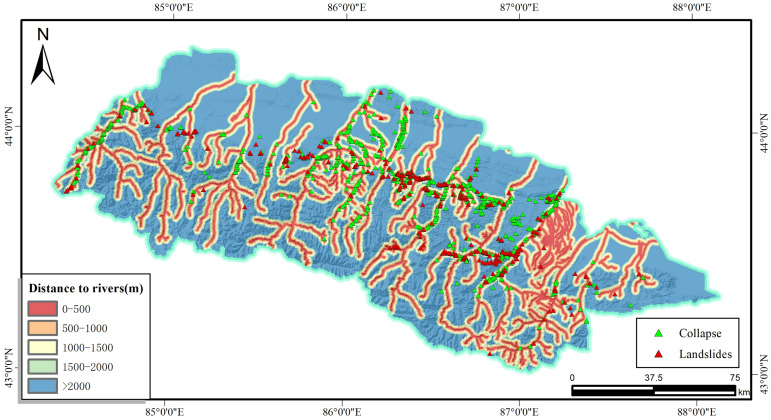
Distance to rivers and disaster distribution map.

Therefore, to facilitate the statistics, a buffer zone was established for the analysis at 500 m intervals centred on the river using the GIS platform. As shown in Fig 2, the buffer zone is divided into 0–500 m, 500–1000 m, 1000 m-1500 m, 1500 m-2000 m and more than 2000 m. Further statistics show that there are 692 slope hazards in the range of 500 m from the river, which accounts for 59.30% of the total number of hazards in the study area. In addition, as the distance from the river increases, the number of slope hazards decreases.

#### 2.3.2. Topography and landform.

**Fig 3:**
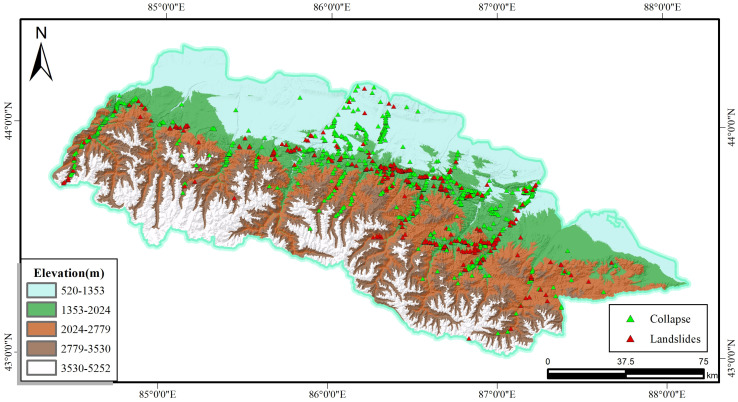
Elevation and disaster distribution map.

Topography and landform crucially affects the formation [21], distribution, and development of the landslide, which covers the elevation, slope, aspect, curvature, terrain ruggedness extracted from the DEM. Elevation affects the change in potential energy at failure and landslide hazard sites [22], as shown in Fig 3, the elevation ranges from 520 m to 5252 m, with a maximum elevation difference of 4000 m. Using ArcGIS software, elevations were graded into five categories using the natural breakpoint method.

**Fig 4:**
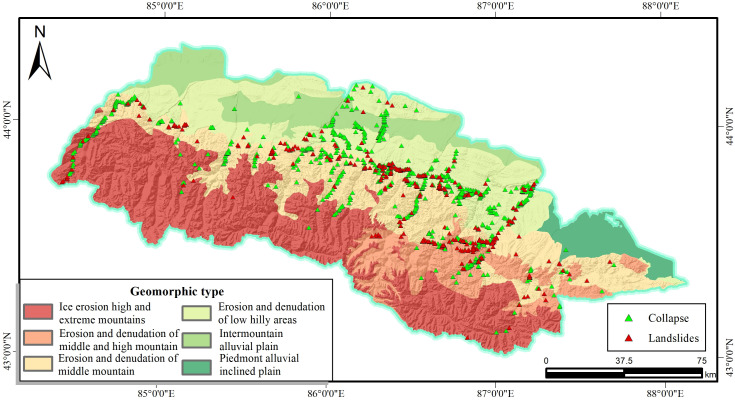
Geomorphic type and disaster distribution map.

As shown in Fig 4, the Landform are categorized into six types: the glacial high and high mountains (Type 1); the erosion and denudation mid-mountains (Type 2); the intermountain alluvial plains (Type 3); the erosion, the denudation high and mid-mountains (Type 4); the piedmont alluvial inclined plains (Type 5); the erosion, denudation low hills and ridges (Type 6). Approximately 79% of the slope disasters occurred in the mid-mountain areas with low hills and ridges rather than the flat terrain. Although the extremely high mountains and mountainous areas have the material basis conditions for slope disasters, the transportation in this area is inconvenient, and even snow accumulates all year round, with few people visiting and very few threats.

**Fig 5:**
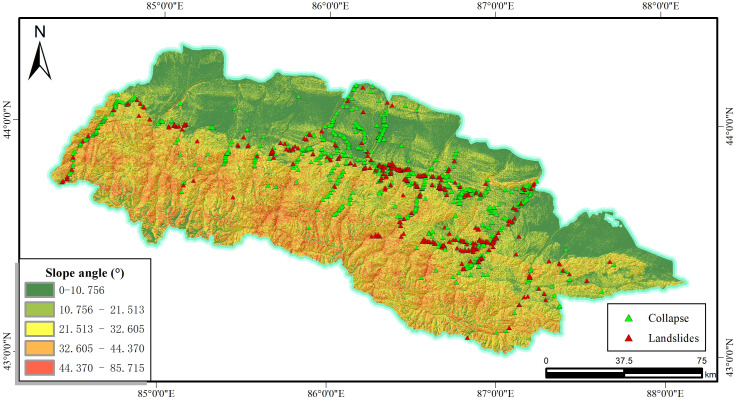
Slope angle and disaster distribution map.

The slope angle influences the movement of soil or rock subjected to the gravity, which predisposed the occurrence of slope disasters, as illustrated in Fig 5. Slope factors are graded using the natural breakpoint grading method, which is divided into 5 levels.

**Fig 6:**
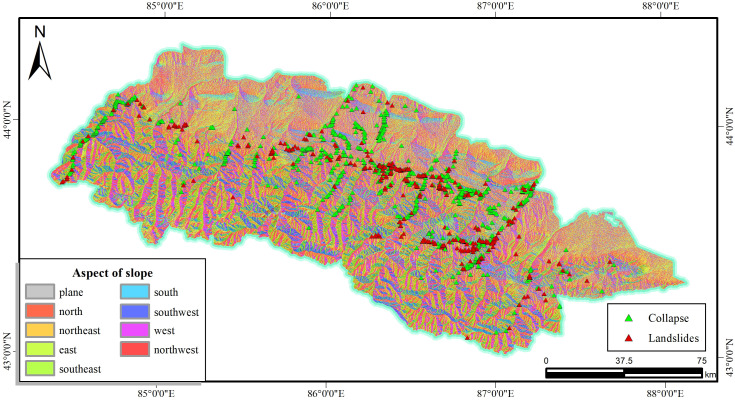
Aspect of slope and disaster distribution map.

As depicted in Fig 6, the slope aspect generally affects both the water and the thermal conditions, which works on the plant growth, the distribution of natural landscapes, human settlements, and economic activities layout in turn. The slope aspect factor is graded according to the actual situation and is divided into eight categories, namely: east, west, south, north, south-east, south-west, north-east, north-east and north-west.

**Fig 7:**
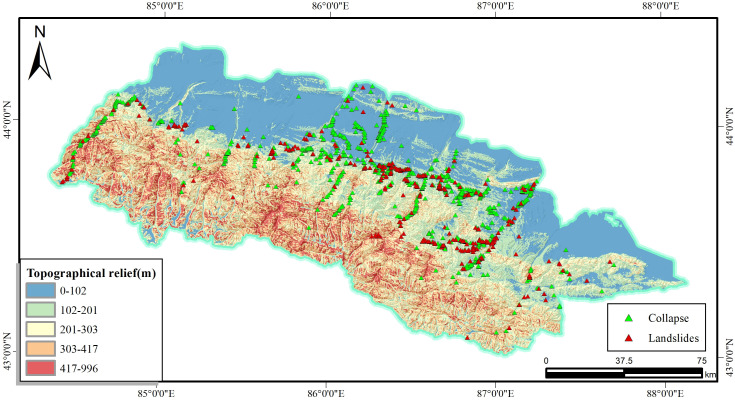
Topographical relief and disaster distribution map.

The ruggedness, also named as the relative height difference, will affects the accumulation of loose materials, which exacerbates the instability and collapse of the slope, even the occurrence of the landslide, as illustrated in Fig 7. The terrain relief factor is graded using the natural breakpoint grading method and is divided into 5 levels.

**Fig 8:**
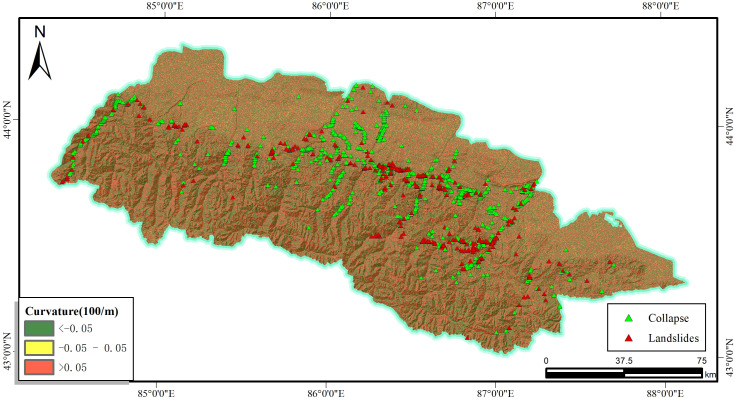
Curvature and disaster distribution map.

The positive curvature of the slope is for the convex while the negative is for the concave, the value of which closer to zero represents the smoother terrain. In general, the curvature affects the accumulation and drainage of water, which also impacts the stability of soil or rock slopes (see Fig 8). In the paper, areas with curvature values between −0.05 and 0.05 are considered as flatter areas and other areas as undulating areas, with a total of three classes.

#### 2.3.3. Human engineering activities.

**Fig 9:**
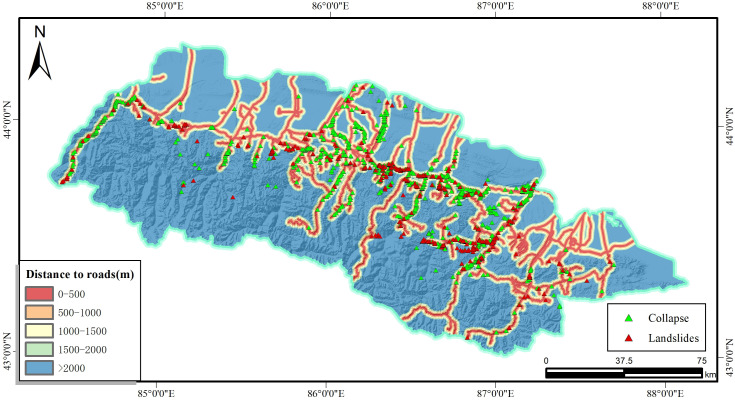
Distance to roads and disaster distribution map.

Roads are an important link to promote regional economic development and enhance regional exchanges, however, a series of human activities such as excavation of the foot of slopes for the construction of roads have changed the original stress state of the slopes. In general, the closer to the highway, the greater the disturbance to the geological environment [23]. In order to facilitate the statistics, the study area was set up as a multi-ring buffer every 500m in the GIS platform to analyse the road factor. As shown in Fig 9, there are five groups, which are 0–500 m, 500–1000 m, 1000 m-1500 m, 1500 m-2000 m and >2000 m. The statistical results show that there are 895 slope hazards occurring within 500 m from the highway, which accounts for 76.69% of the total number of hazards. Therefore, the distance from the road is also determined as one of the evaluation indicators.

**Fig 10:**
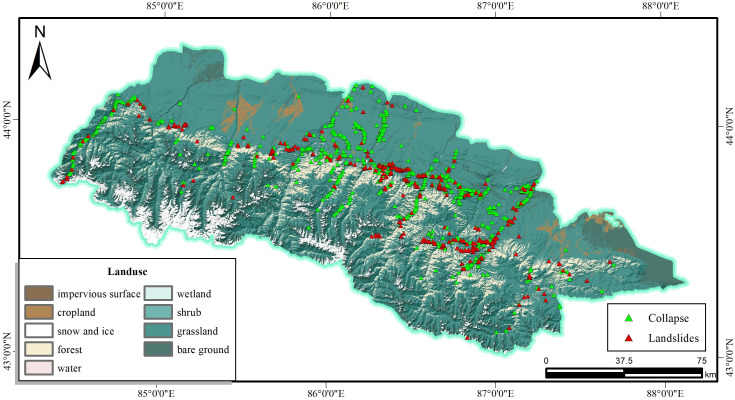
Landuse and disaster distribution map.

The land use pattern is closely related to human activities, which also affects the stability of slopes to a certain extent. The study area has rolling hills, rugged peaks and lush vegetation, and the land use type factor is graded according to the actual type, which is divided into 9 categories, namely: cropland, forest, shrub, grassland, water, snow and ice, bare groud, and Imperxious surface, Wetland. where the main land use patterns are grassland, woodland and bare land. As can be seen from Fig 10, grassland accounts for 69.44 per cent of the total area of the study area, with 1050 slope failures, a proportion of 89.12 per cent.

#### 2.3.4. Geological environment.

**Fig 11:**
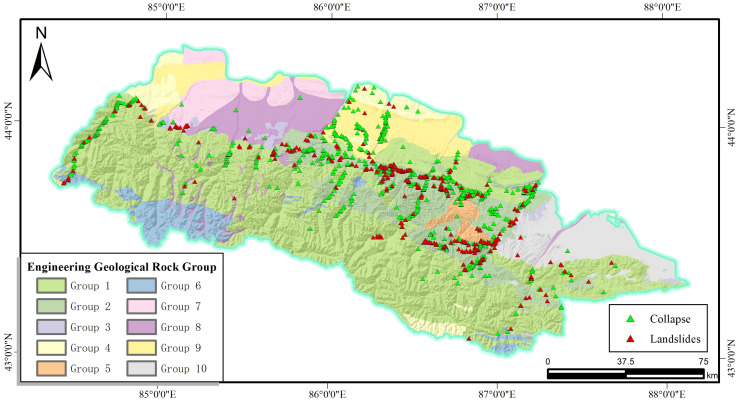
Engineering geological rock group and disaster distribution map.

The engineering geological rock group is one of the main factors affecting slope stability. The categories of rock groups in the study area are divided into three major categories: clastic rocks, magmatic rocks and metamorphic rocks. On the basis of the risk census data of the whole territory, combined with the information collected by the risk assessment project in each county and municipal area for refinement, the article subdivided the engineering geological rock groups in the study area into 10 types, as shown in Fig 11.

Among them, the clastic rock group includes laminated and massive hard and harder carbonate rock sandwiching clastic rock group dominated by greywacke, siltstone and tuff (Group1); interbedded, laminated hard – harder clastic rock group dominated by sandstone, mudstone and conglomerate (Group2); interbedded harder – softer clastic rock group (Group3); and interbedded softer sandstone, conglomerate, mudstone dominated clastic rock group (Group4). The metamorphic rocks are hard – harder layered and blocky metamorphic rocks (Group5). Igneous rocks are blocky, hard, magmatic rock groups (Group6). Soils in the study area are divided into four classifications: special soils (wind-accumulated deserts) (Group7); gravelly monolithic soils (Group8); loess and pebble-gravel bilayered soils (Group9); and sandy, chalky cohesive, and sandy-gravelly multilayered soils (Group10). Statistically, the interbedded, laminated hard – harder to sandstone, mudstone, conglomerate-based clastic rock group distributes the most slope hazards, accounting for 57.84 per cent of the total hazards.

**Fig 12:**
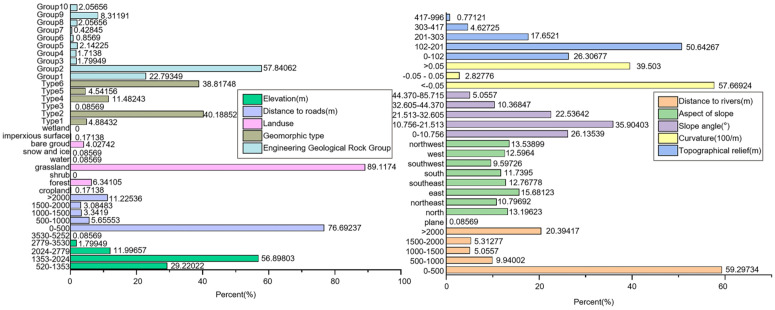
The frequency ratio of landslides in the graded evaluation indicators.

Fig 12 shows the distribution of failure and landslide hazards in the hierarchy of evaluation factors.

### 2.4. Methodology

**Fig 13:**
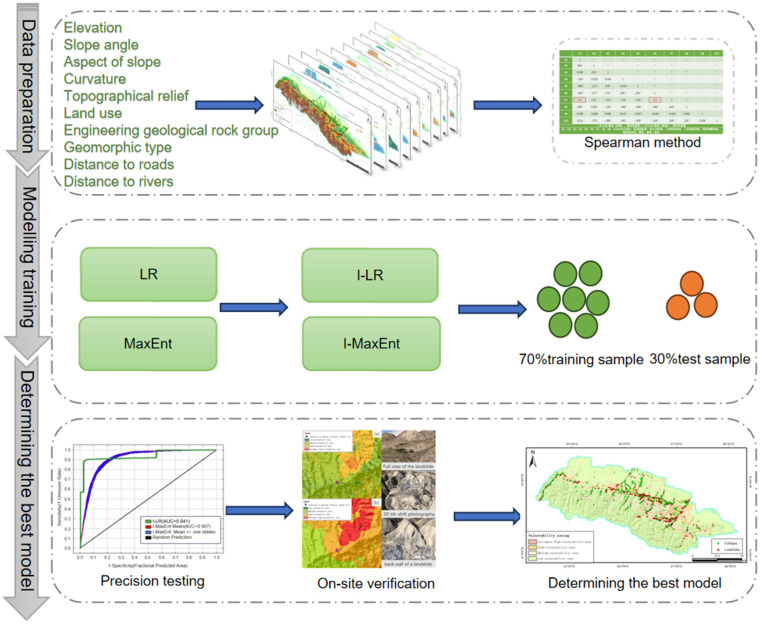
Technical routs for this research.

As depicted in Fig 13, the Spearman’s correlation coefficient analysis were conducted via the SPSS software for preliminary testing to eliminate these strongly correlated factors from these aforementioned evaluation indicators before the establishment of the final assessment system. In order to obtain the susceptibility zoning map, 1167 points were randomly selected as negative samples within the non-susceptible zones using ArcGIS according to the information value method. Note that the minimum distance between these random points is one kilometer and the ratio between the training sample and the validation sample is 7:3. The values of each evaluation factor (I) are incorporated into the Maximum Entropy model and the Binary Logistic Regression model to generate the coupled machine learning models, namely the I-MaxEnt model and the I-LR model. The accuracy of the model predictions in terms of the landslide susceptibility assessment in the southern mountainous areas of the Northern Tianshan Economic Belt is verified through the ROC curves. The optimal model is then determined based on the field validation.

#### 2.4.1. Statistical methodology – Information value method.

The information value (I) is a useful metric to reflect the predictive power of factors [24]. In practice, various geological factors generally play different roles and a higher information value always indicates a higher likelihood of the geological disasters occurrence. The information value of the evaluation indicator can be calculated by Equation (1):


$$I=\sum_{i=\text{1}}^{n}I\left(x_{i},H\right)=\sum_{i=\text{1}}^{n}ln\frac{N_{i}/N}{S_{i}/S}$$ (1)

where, S represents the total area of the study area; represents the total area of geological disasters; N is the total number of geological disaster; stands for the total number of geological disaster points distributed within a given evaluation indicator. Note that the information value can be either the positive or the negative, for which the positive value indicates a higher landslide susceptibility, while the negative value indicates a lower likelihood of slope disasters.

#### 2.4.2. Machine learning models- logistic regression (LR) model.

Logistic regression model is of effectiveness when the relationship between a binary dependent variable and independent variables is requested. Based on the linear regression for multivariate statistical analysis, the computation process of LR model is convenient with its high accuracy. As one of the most popular method applied for the landslide susceptibility assessment, the occurrence of landslide is generally determined as the dependent variable and the evaluation indicators act as the independent variables [25,26]. With the application of the SPSS, the values of the P will be then calculated [19]. Generally, the following equation is adopted to establish the LR model:


$$\left\{ \begin{array}{c} P(Y=\text{1}|X)=\frac{\text{1}}{\text{1}+e^{-Z}}\\ Z=\beta_{\text{0}}+\beta_{\text{1}}x_{\text{1}}+\beta_{\text{2}}x_{\text{2}}+...+\beta_{n}x_{n} \end{array}\right.\;$$ (2)

in which, the value of P is the probability of the landslide occurrence, which ranges from 0 to 1. represents the coefficient of the logistic regression model.

#### 2.4.3. Machine learning models-maximum entropy (MaxEnt) model.

To accurately assess the landslide susceptibility, the Maximum Entropy (MaxEnt) model initially developed by Phillips et al. was adopted in the present research [27]. Compared to other machine learning models, the MaxEnt model requires limited data but with a high prediction accuracy. By inputting the real location of the landslide and determined evaluation indicators, the probability distribution of landslide in unknown areas can be obtained via the specific algorithms in the MaxEnt model [27–29]. The application of the MaxEnt model for landslide susceptibility assessment enables the quantitative analysis of the impact of slope disasters, which is beneficial for the distribution of the landslides [30]. The MaxEnt model is expressed by the following equation:


$$H\left(Y|X\right)=-\sum_{i=\text{1}}^{n}P\left(x_{i}\right)\sum_{j=\text{1}}^{m}P\left(y_{j}|x_{i}\right)\text{log}p(y_{j}|x_{i})$$ (3)

where, X represents the environmental condition, and Y represents the probability of slope hazards.

#### 2.4.4. Coupled model.

The evaluation samples for the coupled model of the information value (I) model and the machine learning models are the landslide disaster points and an equal number of non-disaster points, by extracting the I values of various evaluation factors at multiple levels. The contribution weights of evaluation indicators and the intervals are all taken into accounted by the coupled model. The basic idea behind the coupled model is to utilize the I values of each evaluation indicator as the input variables for both the logistic regression (LR) model and the Maximum Entropy (MaxEnt) model.

### 2.5. Accuracy assessment

The Receiver Operating Characteristic (ROC) curve is widely applied to evaluate the performance of the theoretical model by comparing the cumulative percentage of geological disasters under the given conditions [31]. The ROC curve is evaluated based on how close it is to the upper left corner, with a closer distance indicating higher model accuracy. In practice, the accuracy can be evaluated by the overall area under the curve (AUC). The larger values of the AUC generally indicate a higher accuracy and better precision of the model. If AUC < 0.6, it indicates poor accuracy and if 0.6 ≤ AUC < 0.7, it represents the fair accuracy. When 0.7 ≤ AUC < 0.8, it is of good accuracy, while 0.8 ≤ AUC < 0.9, it indicates a very good accuracy. If AUC ≥ 0.9, it means an excellent accuracy [32]. To enhance the authenticity of the model, on-site field validation was incorporated as a crucial means to verify model accuracy, aiming at determining the most suitable model for landslide susceptibility assessment in the southern mountainous region of the economic belt on the northern slope of the Tianshan Mountains.

## 3. Results

### 3.1. Rigorous analysis of multicollinearity

#### 3.1.1. Correlation analysis.

Considering that the evaluation index will directly affect the accuracy of the model, the correlation coefficient (R) between the factors was calculated based on SPSS software using the Spearman method before the experimental test. Unlike the Pearson correlation coefficient, the Spearman coefficients are calculated using the rank (i.e., ordering) of the variables, rather than using raw numerical data. Discrete values can be converted to ordinal numbers for ranking based on type. The value of Spearman’s coefficient ranges from −1–1, where −1 indicates complete negative correlation, 1 indicates complete positive correlation, and 0 indicates no correlation. In general, |R| < 0.3 is regarded as weak correlation; 0.3 ≤ |R| < 0.5 is regarded as low correlation; 0.5 ≤ |R| < 0.8 is regarded as significant correlation; 0.8 ≤ |R| < 1 is regarded as high correlation; and |R| = 1 is regarded as linear correlation [33]. In this study, intervention was required when the threshold value of 0.5 was exceeded. The analysis shows that there is a high correlation between the degree of terrain undulation and elevation as well as the type of topography and geomorphology, so the terrain undulation is excluded from the evaluation index system. In other words, the evaluation indicators used for landslide susceptibility assessment include elevation, distance from roads, distance from water bodies, land use type, engineering geological rock group, topographic landform type, slope, curvature and slope direction. The correlation test of the evaluation indicators is shown in Table 2.

**Table 2:** Correlation test of evaluation indicators.

	X1	X2	X3	X4	X5	X6	X7	X8	X9	X10
X1	1									
X2	0.087**	1								
X3	−0.012	0.257**	1							
X4	0.099**	−0.048	0.089**	1						
X5	−0.170**	0.074*	0.116**	−0.005	1					
X6	0.053	−0.015	0.152**	0.208**	0.193**	1				
X7	0.623**	−0.054	−0.127**	−0.093**	−0.333**	−0.508**	1			
X8	0.004	−0.018	−0.196**	−0.041	−0.086**	−0.220**	0.242**	1		
X9	0.047	0.024	−0.038	0.027	0.051	0.055	−0.066*	−0.085**	1	
X10	−0.011	−0.065*	−0.067*	−0.069*	−0.117**	−0.149**	0.105**	0.091**	−0.044	1

*. Significant at the 0.05 level (two-tailed); **. Significant at the 0.01 level (two-tailed).

X1, X2, X3, X4, X5, X6, X7, X8, X9, X10 are elevation, distance to roads, distance to rivers, land use, engineering geological rock group, geomorphic type, topographic relief, slope angle, curvature, and aspect of slope, respectively.

#### 3.1.2. Variance inflation factor calculation.

The presence of statistical multicollinearity among these evaluation indicators may results in distorted estimation and thus it is crucial to introduce the Variance Inflation Factor (VIF) and tolerance into the analysis prior to the application of the I-LR model. based on the analysis by using SPSS, these evaluation indicator should be removed either if the tolerance value is less than 0.1 or the VIF value exceeds 10 [34]. As listed in Table 3, the minimum values of the tolerance is 0.317, and the maximum vaules of VIF is 3.154, suggesting that there is no strong multicollinearity between these determined evaluation indicators for the landslide susceptibility assessment.

**Table 3:** Evaluation factor covariance diagnostics.

Evaluation factors	Tolerances	VIF
Engineering geological rock group	0.847	1.180
Land use	0.875	1.143
Distance to rivers	0.691	1.447
Curvature	0.995	1.005
Aspect of slope	0.980	1.021
Slope angle	0.699	1.431
Geomorphic type	0.450	2.221
Distance to roads	0.518	1.931
Elevation	0.317	3.154

### 3.2. Information value calculation

Environmental factors was reclassified via the ArcGIS natural break classification method and the “Extract Values To Points” tool was adopted to get the disaster distribution at different classification levels. Subsequently, the Information Value (I) model was applied to obtain the corresponding values, the results of which are presented in Table 4.

**Table 4:** Information value (I) calculation results.

Evaluation indicator	Hierarchy of factors	Number of disasters	Information value
Distance to rivers (m)	0-500	692	0.597
	500-1000	116	−0.123
	1000-1500	59	−0.357
	1500-2000	62	−0.278
	2000	238	−0.391
Aspect of slope	Plane	1	0.358
	North	154	−0.055
	Northeast	126	−0.140
	East	183	0.027
	Southeast	149	0.085
	South	137	0.077
	Southwest	112	0.002
	West	147	0.009
	Northwest	158	0.017
Slope angle(°)	0-10.756	305	−0.050
	10.756–21.513	419	0.180
	21.513–32.605	263	0.032
	32.605–44.370	121	−0.226
	44.370–85.715	59	−0.229
Curvature(100/m)	<−0.05	673	0.073
	−0.05-0.05	33	−0.087
	>0.05	461	−0.083
Elevation(m)	520-1353	341	0.065
	1353-2024	664	0.450
	2024-2779	140	−0.230
	2779-3530	21	−1.015
	3530-5252	1	−2.262
Distance to roads(m)	0-500	895	0.832
	500-1000	66	−0.215
	1000-1500	39	−0.380
	1500-2000	36	−0.354
	2000	131	−0.759
Landuse	Cropland	2	−0.980
	Forest	74	−0.192
	Shrub	0	−2.262
	Grassland	1040	0.108
	Water	1	−0.863
	Snow and ice	1	−1.719
	Bare groud	47	−0.535
	Imperxious surface	2	0.048
	Wetland	0	−2.262
Geomorphic type	The glacial high and high mountains	57	−0.824
	The erosion and denudation mid-mountains	469	0.258
	The intermountain alluvial plains	1	−1.667
	The erosion, the denudation high and mid-mountains	134	0.059
	The piedmont alluvial inclined plains	53	−0.336
	The erosion, denudation low hills and ridges	453	0.259
Engineering geological rock group	The special soils (aeolian desert)	5	−1.007
	The interlayered clastic rock formations	21	0.152
	Gravel single-layer soils	24	−0.555
	The igneous rock includes igneous rock formations	10	−0.721
	The loess, gravel double-layer soils	97	0.057
	The clastic rock formations are dominated by the Limestone, the dolomite, and the shale	266	−0.327
	The layered sandstone, mudstone, Conglomerate-dominated clastic rock formations	675	0.589
	The soft clastic rock formations dominated by sandstone, Conglomerate, and mudstone	20	−0.361
	The multi-layer soils of sand, loam, sticky soil, and gravel	24	−0.509
	The metamorphic rock category consists of layered and Massive metamorphic rock formations	25	0.204

The overall information value distribution map for landslides in the southern mountainous region of the economic belt on the northern slope of the Tianshan Mountains was generated by the raster calculator to sum up the ratings of the 9 evaluation indicators. This resulting map was then categorized into high, moderate, low, and non-susceptible areas, in which the non-susceptible areas indicates the absence of landslide disasters.

### 3.3. The I-MaxEnt model

**Fig 14:**
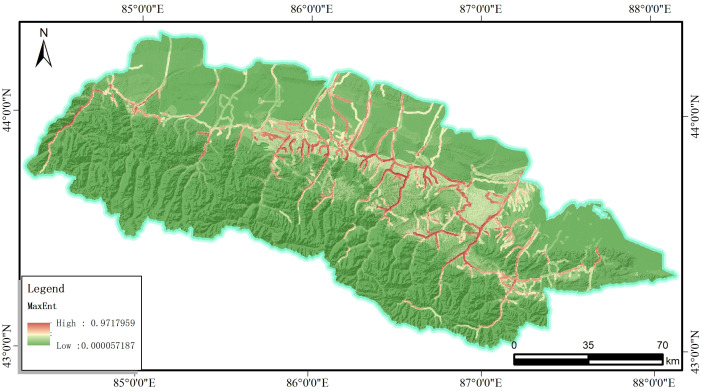
Results of the I-MaxEnt model.

Based on the ‘multi-value extraction to point’ function of ArcGIS software, the information value corresponding to the raster data value of the selected evaluation factors was extracted to 2334 sample points, constituting the evaluation factor data table. The exported EXCEL data table was then imported into MaxEnt software. In the process of basic parameter setting, 70% of the point information is randomly selected to generate the training model, and the remaining 30% of the point information is left for validation to obtain the susceptibility evaluation map under the I-MaxEnt model, as shown in Fig 14. In practice, a Random seed and the Subsample methods are adopted together to validate the model with a Bootstrap number of 10 times. After the generation of the response curves for each evaluation indicators, the jackknife [35]method will be employed to assess the contribution of each indicator.In practice, a Random seed and the Subsample methods are adopted together to validate the model with a Bootstrap number of 10 times.

#### 3.3.1. Assessment by the Jackknife method.

**Fig 15:**
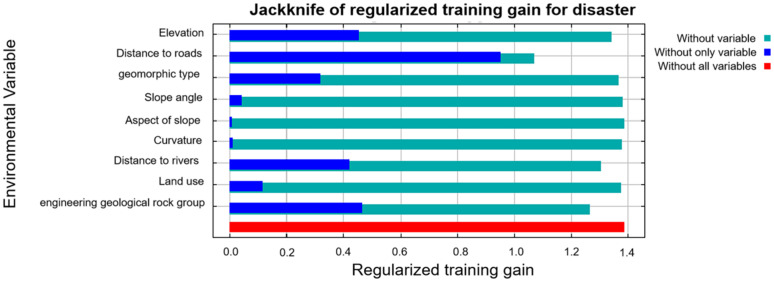
Jackknife inspection diagram.

It is not easy to completely eliminate the correlations between different evaluation indicators. The jackknife method is thus employed to assess the contribution of each indicator, aiming at determining the most important indicator in the model. As can be seen from Fig 15, the contribution of the distance to road is most obvious, followed by the engineering geological rock group, elevation, distance to river, and the terrain landform type. Compared to their counterparts, land use pattern and slope angle are moderately important, while the influence of the aspect and the curvature is not obvious.

#### 3.3.2. The single-factor response curves.

**Fig 16:**
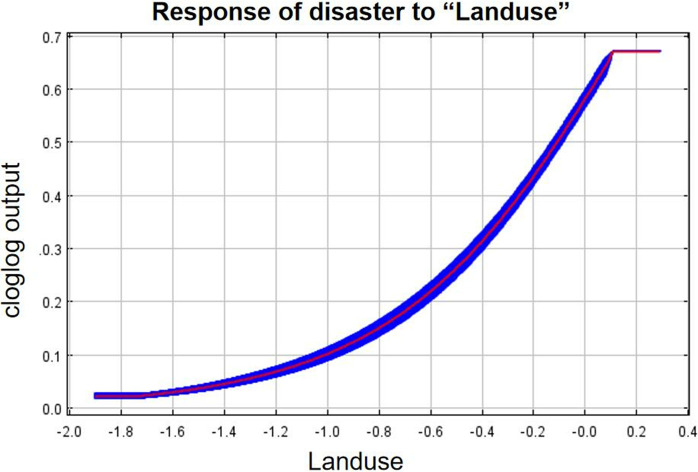
Response of disaster to “Landuse”.

**Fig 17:**
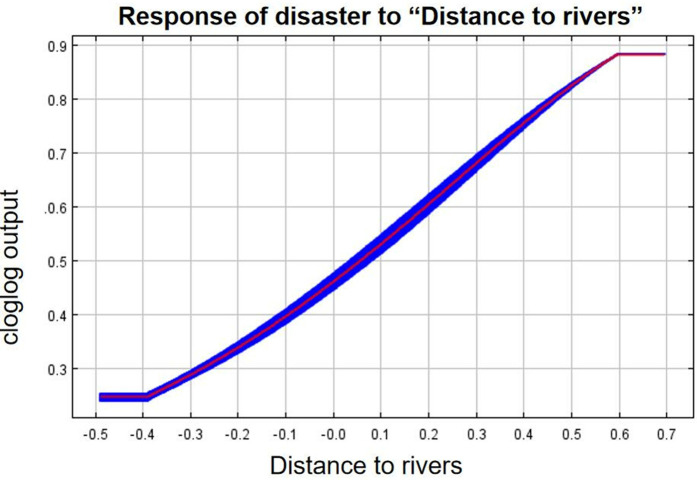
Response of disaster to “Distance to rivers”.

**Fig 18:**
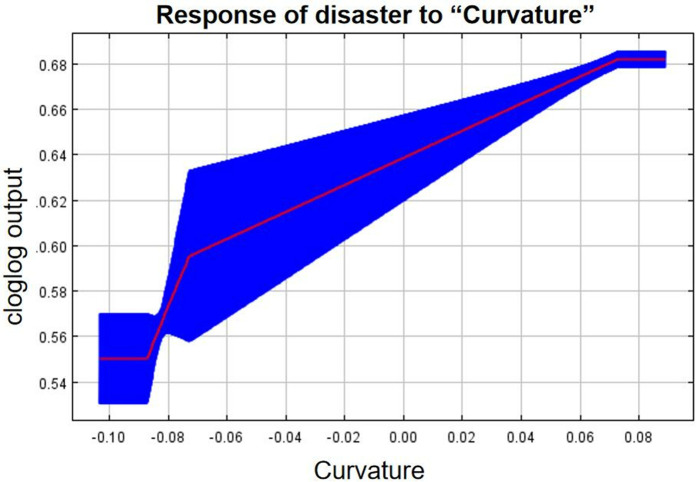
Response of disaster to “Curvature”.

**Fig 19:**
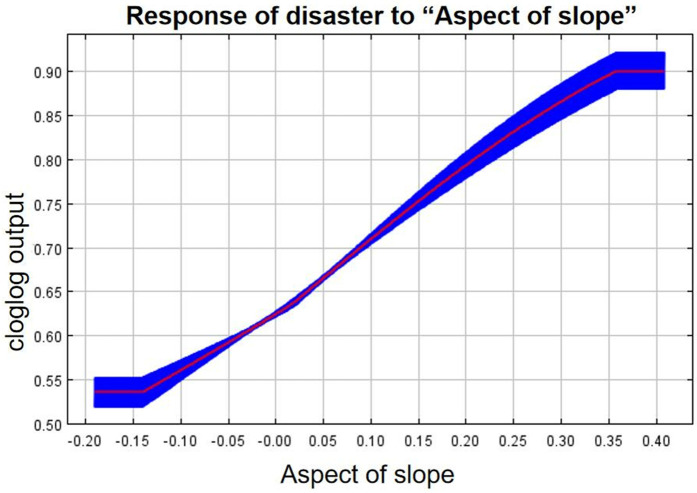
Response of disaster to “Aspect of slope”.

**Fig 20:**
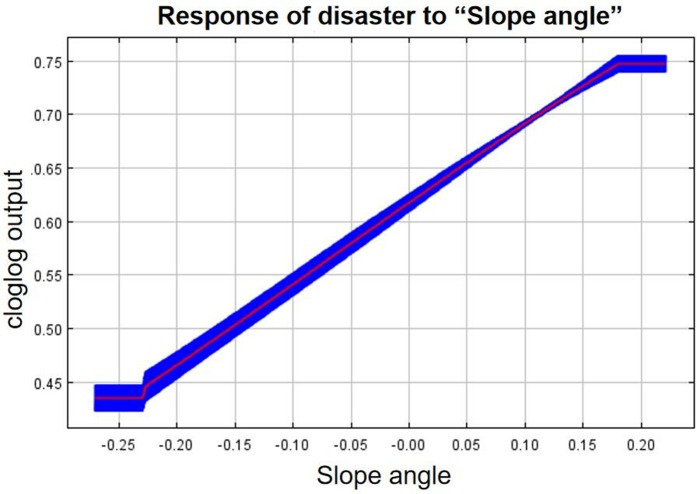
Response of disaster to “Slope angle”.

**Fig 21:**
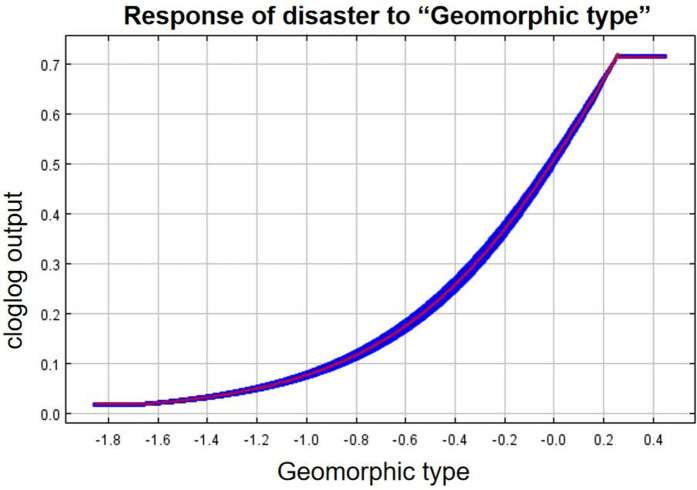
Response of disaster to “Geomorphic type”.

**Fig 22:**
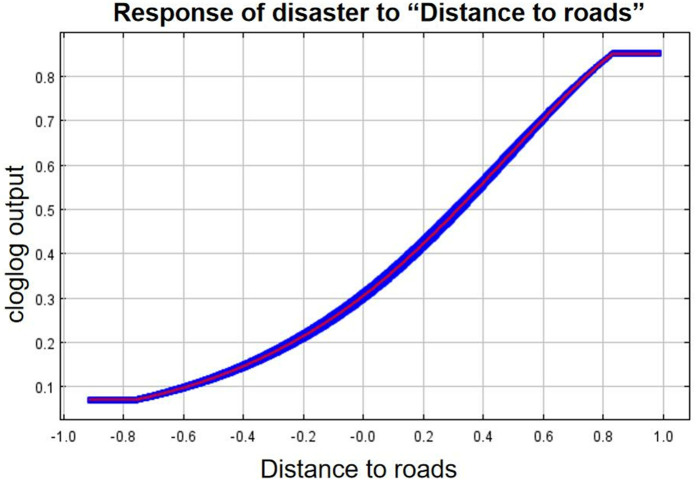
Response of disaster to “Distance to roads”.

**Fig 23:**
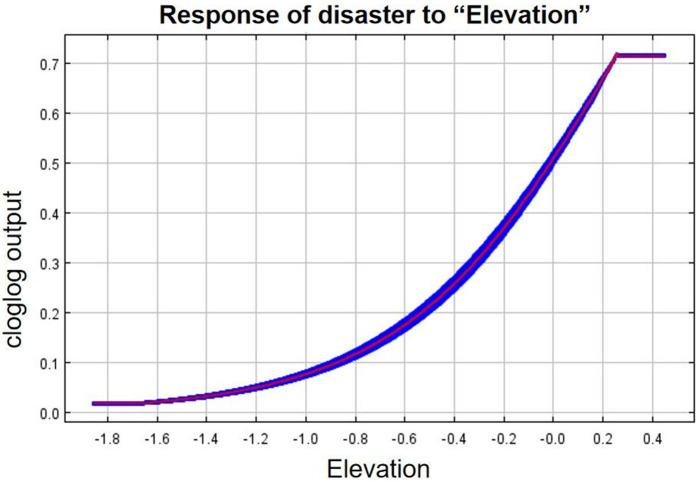
Response of disaster to “Elevation”.

**Fig 24:**
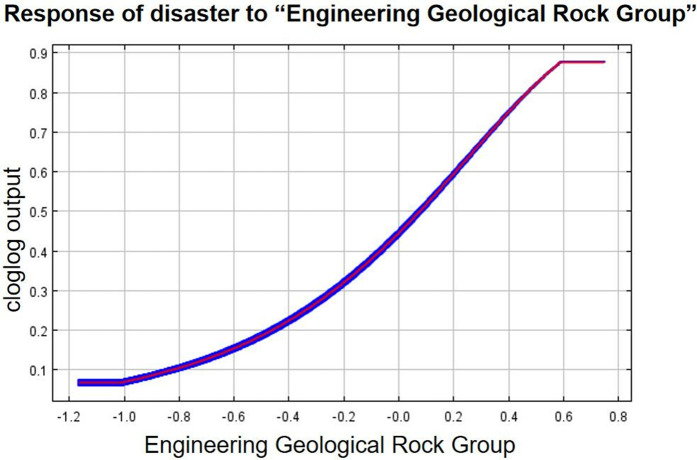
Response of disaster to “Engineering Geological Rock Group”.

As can be seen from the single-factor response curves (Figs 16–24), combining the information value model with the MaxEnt model allows the features of the information value model to be incorporated into the coupled model. The likelihood of landslide is closely related to their respective Information Value. In other words, the larger Information Value always corresponds to the higher probability of landslides.

### 3.4. Information value-logistic regression model (I – LR Model)

#### 3.4.1. Selection of non-disaster points.

**Fig 25:**
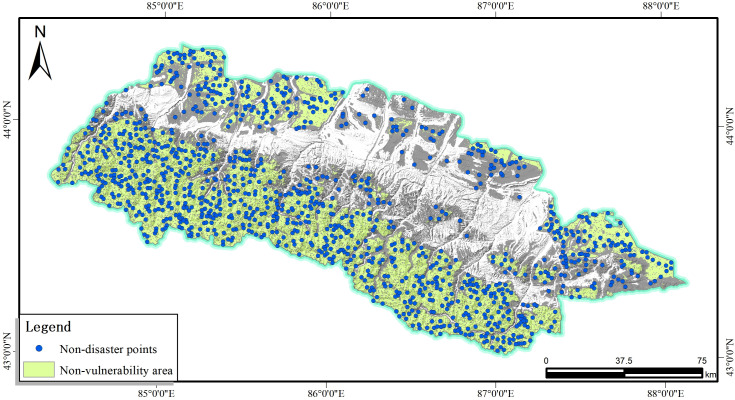
Layout of non-disaster points.

The selection of non-disaster points and the efficient handling of data on hazard-causing factors are key aspects of the evaluation of the susceptibility to geological hazards. It involves many aspects such as data collection, integration, analysis and management. Geographic Information System (GIS) plays a crucial role in this process, and GIS software such as ArcGIS can not only process spatial data and attribute data, but also carry out spatial analyses, which provides powerful technical support for the evaluation of geohazard susceptibility.When the logistic regression model is adopted to conduct the landslide susceptibility assessment, it is necessary to construct non-disaster points within the non-susceptible areas.The distribution of non-disaster sites is shown in Fig 25. Except for the 1:1 ratio between these disaster and non-disaster points, the distance between non-disaster points and disaster points, as well as the distance between any two non-disaster points should be greater than 1000 meters. These three criteria were utilized to generate non-disaster points in the ArcGIS platform, in which the disaster and non-disaster points are assigned values of 1 and 0, respectively. Notice that these sample points were randomly divided into training and testing sets with a constant ratio of 7:3.The distribution of non-disaster sites is shown in Fig 25.

#### 3.4.2. Results from the I-LR coupled model.

The I-LR coupled model is based on SPSS will be extracted 2334 sample points with I values of 9 types of influencing factors as independent variables and landslide hazard points as dependent variables. In the I-LR coupling model with binary as the dependent variable, the 2334 sample points and their respective evaluation index information values (I) were used as independent variables, and these slope hazard points were coded as 1, while these non-slope hazard points were coded as 0. Based on the ArcGIS software, the selected evaluation factor information values were extracted into 2334 sample points with the function of ‘Multi-value Extraction to Points’. Based on ArcGIS software, the selected values of evaluation factors were extracted to 2334 sample points by using the function of ‘multi-value extraction to points’. Then the extracted data table was input into SPSS software to get the regression coefficient (B) value and its related significance.

Note that only if the significance levels (Sig) is below 0.05, the landslide susceptibility assessment is meaningful. However, the values of Sig for curvature factor was 0.192. Failed to pass the significance test, this indicator have to be removed from the evaluation system. The revised analysis is then performed with other 8 remained indicators, the results of which are listed in Table 5. It is apparent in Table 5 that all Sig values are below 0.05, suggesting that all these 8 indicators passed the statistical significance test within an acceptable error margin.

**Table 5:** Logistic regression coefficients and significance tests.

	Constant	Engineering geological rock group	Land use	Distance to rivers	Aspect of slope	Slope angle	Geomorphic type	Distance to roads	Elevation
B	2.666	3.793	1.704	3.915	3.518	3.703	3.106	3.581	1.700
Sig	<0.01	<0.01	0.01	<0.01	0.044	<0.01	<0.01	<0.01	<0.01

**Fig 26:**
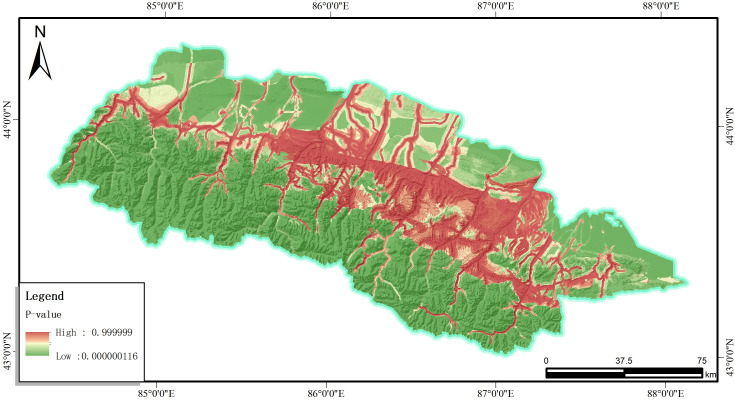
Distribution P values calculated by the I-LR model.

It is obvious that all these eight factors exhibit positive regression coefficients and the weights of which ranked from highest to lowest are listed in below: the distance to river, Engineering geological rock group, Slope angle, Distance to roads,Aspect of slope, geomorphological type, Land use type, and Elevation. According to the obtained regression coefficients of each factor, combined with the formula (2) using GIS technology to superimpose the layers according to the coefficients, the probability of the occurrence of slope hazards in the mountainous areas in the southern part of the economic zone of the northern slopes of the Tianshan Mountain P map was calculated. The distribution of P-values is shown in Fig 26.

### 3.5. The landslide susceptibility assessment

**Fig 27:**
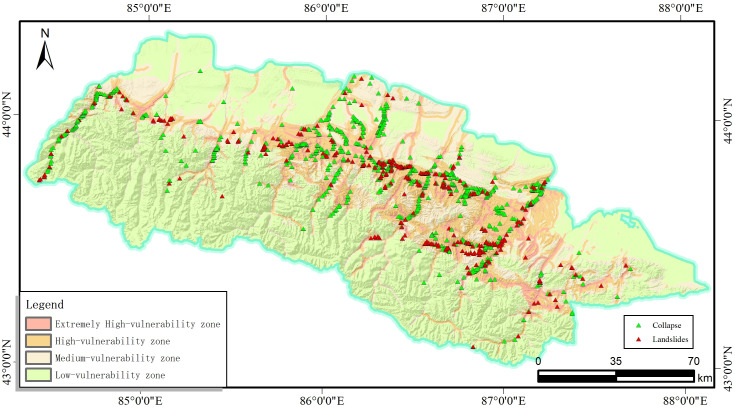
The I-LR model landslide susceptibility assessment results.

**Fig 28:**
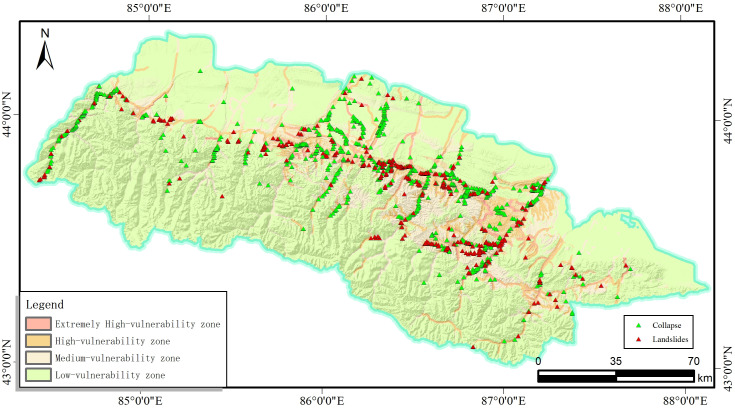
The I-MaxEnt model landslide susceptibility assessment results.

The results obtained from the I-LR model and I-MaxEnt model were imported into the ArcGIS software to get the landslide susceptibility map(Figs 27, 28). Herein, four classification zones (i.e., very high, high, moderate, and low susceptibility) are marked on the map as per the specific standards for different models. notice that these zones with similar values are grouped together and these values with significant differences are placed at the boundaries.

It is evident from Figs 27 and 28 that these two coupled models exhibit distinct characteristics although there are still something of similarity, which are listed in below for reference:

(1) Extremely high risk areas are distributed near rivers and roads, which is mainly attributed to effects of artificial construction activities, such as the excavation and embankment dumping on the original geological environment Moreover, water bodies can often soften and erode the slopes along the river associated with the reduced strength of the rock mass, which will facilitate the occurrence of landslide in turn.
(2) The extremely high and highly prone areas are mainly distributed in the middle and high mountains and hilly areas of the study area. Among them, the high-prone area is mainly located in Changji City, Hutubi County and Manas County. The locations of high and medium prone areas predicted by the two models are roughly the same, but the areas are different. The areas of high and medium prone areas predicted by the I-LR model are higher than those predicted by the I-MaxEnt model.
(3) Although the area of the very high landslide susceptibility zone in the Miaoergou township in Changji is approximately equal, there is still a notable disparity in the area classified as the high susceptibility zone. That is, this area falls into the high susceptibility zone as predicted by the I-LR model, whereas it is marked as the moderate susceptibility zone with the application of the I-MaxEnt model.

As can be seen from Table 6, the I-LR model and I-MaxEnt model gradually increase the number of disaster points and decrease the distribution area within the classification interval as the susceptibility level of landslide disasters increases. This is because as the susceptibility increases, the conditions for triggering landslides within the region are more easily met. For example, the stability of rock and soil masses decreases, and the terrain slope approaches the critical value, thus leading to an increase in the number of potential disaster points. The reduction in the distribution area is due to the more significant clustering of disaster-causing factors in high-susceptibility areas, rather than a widespread, discrete distribution. According to statistics, the total proportion of landslide disaster in the I-LR and I-MaxEnt models falling into the extremely high and high-prone areas is 95.03% and 86.63%, respectively. More than half of the landslide disaster points are developed in the prone areas, while the number of landslide disaster points in the medium-low prone areas is significantly reduced. In accordance with the actual development of landslide disaster in the southern mountainous area of the Tianshan North Slope Economic Belt, both the information coupling models can effectively evaluate the susceptibility of landslide disaster in the study area.

**Table 6:** Coupled model susceptibility partitioning statistics.

Hierarchy	I-LR				I-MaxEnt			
	Number of slope disasters	Percentage of slope disasters (%)	Area (km^2^)	Area proportion (%)	Number of slope disasters	Percentage of slope disasters (%)	Area (km^2^)	Area proportion (%)
Extremely high	970	83.12%	2726.41	12.75%	673	57.67%	804.16	3.76%
High	139	11.91%	3239.87	15.15%	338	28.96%	2071.70	9.69%
Medium	34	2.91%	3253.10	15.21%	109	9.34%	3042.74	14.23%
Low	24	2.06%	12161.84	56.88%	47	4.03%	15462.62	72.32%
Total	1167	100.00%	21381.22	100.00%	1167	100.00%	21381.22	100.00%

### 3.6. Performance evaluation

#### 3.6.1. Accuracy evaluation.

**Fig 29:**
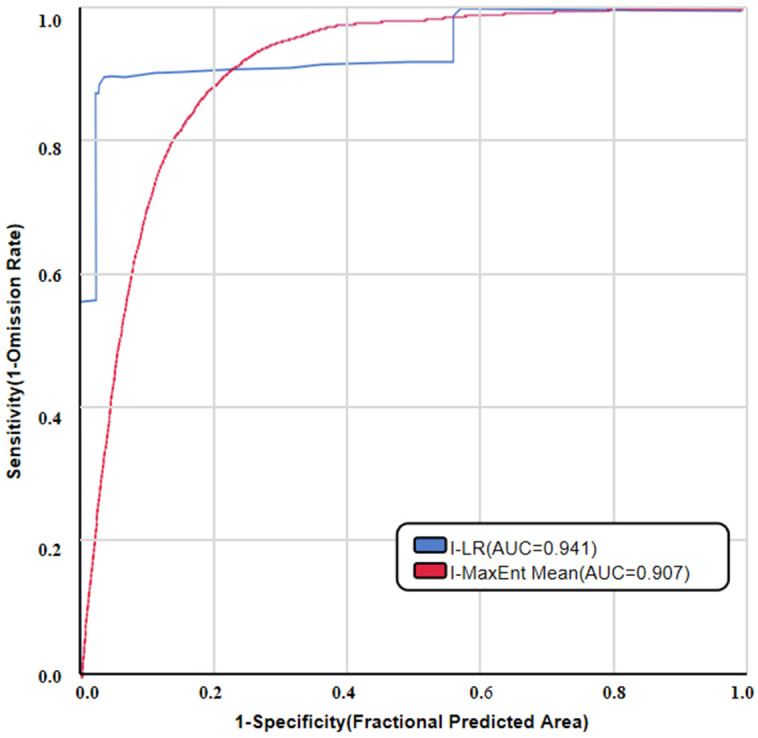
Curves of ROC-AUC.

With the application of the AUC value, the red line of the ROC curve (see Fig 29) represents the training data, while the blue area represents the standard deviation of the testing data. Herein, the black line stands for the random prediction [36]. It can be seen that the values of the AUC of the I-LR and I-MaxEnt models are 0.941 and 0.907, respectively. Although these two models all had a high accuracy, the I-LR model seems to be superior when it is compared with the I-MaxEnt model.

**Fig 30:**
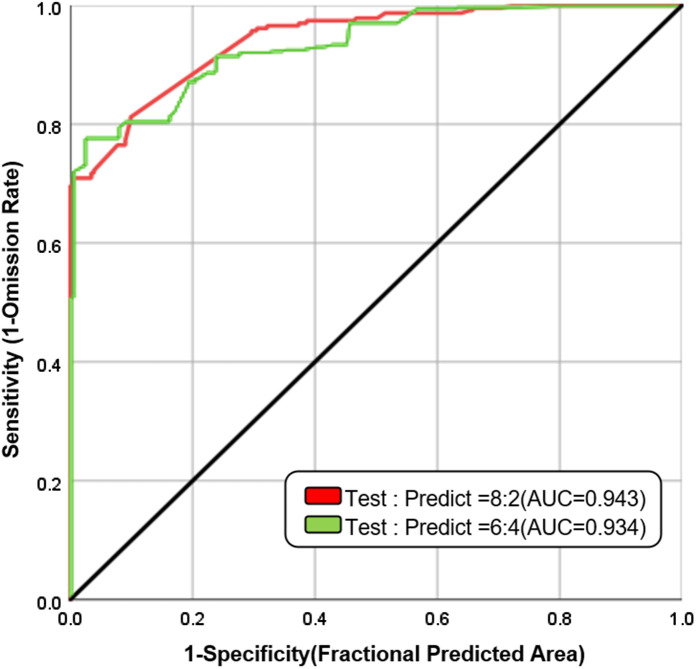
The auxiliary experimental ROC-AUC curve.

The above experiments were carried out when the ratio of experimental data to test data was 7:3. If the ROC accuracy exceeded 0.9, it was necessary to consider whether there was an overfitting issue. Consequently, two additional sets of supporting experiments were added, and the ratio of experimental data to test data was adjusted to 8:2 and 6:4 respectively. As depicted in Fig 30, when the ratio of experimental data to test data was 8:2, the ROC accuracy was 0.943; when the ratio of experimental data to test data was 6:4, the ROC accuracy was 0.934. The results of the three groups were similar, and there was no significant difference, indicating that the experimental results were of high precision and there was no overfitting phenomenon.

#### 3.6.2. Field validation.

The landslide discovered in Miaoergou belonging to Changji was selected for field validation. Through the well excavation and sampling process, it can be seen that the back of the bedrock layer is of a loose structure with developed joint fractures. The length of the landslide ranged from 25 to 180 meters, and the width of which ranged from approximately 20–150 meters. The boundaries, shear outlets, and front edges of the landslide were clearly distinguishable and exhibited a tongue-shaped pattern on the plan view and a convex shape on the profile view. The slope gradient of the landslide ranged from approximately 22–65 degrees. A raised mound with a height of approximately 2–20 meters was formed at the front edge of the landslide. The composition of the material mainly consisted of loess, fragmented stones, and boulders. The back wall of the landslide was steep and almost vertical, with the upper part of the slope experiencing ground cracking and shedding, erosion of the vegetation cover in the middle, exposure of loess, and the soil being moist, loose, and poorly adhesive. Clear tension and localized shear cracks were observed along the side boundaries and back edge. The triggering factors for the landslide included the steep slope gradient at the front edge, the influence of gravity, rainfall, snowmelt infiltration, artificial slope cutting, and earthquakes. Under these influences, the shear stress exceeded the shear strength of the sliding surface, causing downward movement along this surface and resulting in a high susceptibility to landslides.

**Fig 31:**
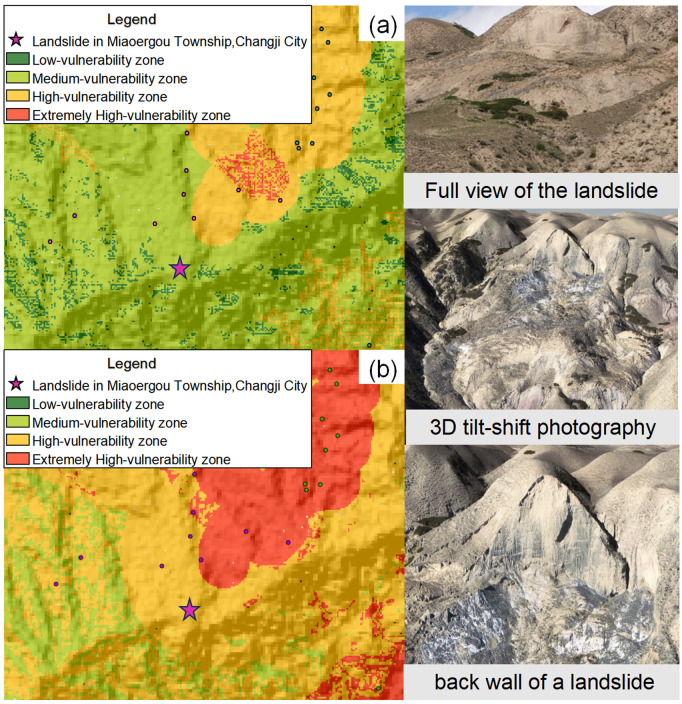
Comparison between field validation and modelling prediction: (a) I-MaxEnt model; and (b) the I-LR model.

It can be observed from Fig 31, that the spatial location of this landslide is situated in the low susceptibility zone predicted by the I-MaxEnt model and the high susceptibility zone predicted by the I-LR model. When the susceptibility level division of the two evaluation results were discussed together, it is apparent that the I-LR model effectively predicted the spatial location of this landslide, which is much more consistent with the actual situation. This observation also suggests that different models can provide different prediction results even at the same research region. By the detailed evaluation on variable models, more accurate and reliable susceptibility assessment could be obtained.

## 4. Discussion

The optimal model accurately assessing the landslide susceptibility is not existed, mainly because that the accuracy of which is closely related to the specific study area. Considering the diversity of predication models, the coupled models including the I-MaxEnt and I-LR theories were adopted in the present research and the accuracy of which was evaluated through the comparison between the ROC curves and regional field validation, respectively. Since that there is no standard for the selection of assessment indicators, 10 evaluation factors were thus chosen accounting for the actual geographical and characteristics of landslide development in the research region.

It is obvious from the I-LR model analysis that the importance of evaluation indicators from high to low level is the distance to river, engineering geological rock group, elope angle, distance to roads, aspect of slope, geomorphological type, land use type, and elevation. Differently, the importance of these assessment indicators are the distance to roads and engineering geological lithology, elevation, distance to river and landform type, when the I-MaxEnt model was adopted. The contribution of the land use and the slope is not significant, while the influence of the aspect and curvature can be ignored. The comprehensive comparison between these two coupled models suggested that the distance to river, distance to roads, and engineering geological lithology are three top indicators affecting the accuracy of landslide susceptibility assessment in this study area. Studies have shown that road construction and river erosion reduce slope stability [37,38]. And there are also large differences in the role of different lithologies in inducing collapse and landslide hazards [39,40]. This observation also agrees well with previous research conducted at the southeastern Nigeria [41–43]. As reported, the slope made of loose soils are more prone to trigger the landslide, when it is compared with the one made of the well-compacted sandstone or the well bonded clay. At the same time, the triggering effect of slope factor on landslides should not be underestimated. In the I-LR evaluation model, the importance of slope factor ranks among the top three, while in the I-MaxEnt model analysis, the gain of slope factor is relatively low. Compared with the results of the two models, the I-LR evaluation model is more reasonable.

Although the LR model exhibited a better performance compared to other statistical models, as verified by previous research in the Enugu State [44,45], it should be noted that the accuracy metrics of the theoretical is also sensitive to exterior surroundings [46]. In the present research, the AUC values of the I-LR model and the I-MaxEnt model were 0.941 and 0.907, respectively. As can be seen from Fig 29, all these two models exhibit a high reliability and the prediction results of which are very similar. Even though, the reason for this similarity, either attributed to the inherent characteristics of the models or the correlation between these evaluation indicators, is still uncertain. Except for the similarity, there are also some differences in between the two models. The first one is that the I-LR model the high susceptibility zone was overestimated associated with the underestimated moderate susceptibility zone. In contrast, the moderate susceptibility zone was slightly overestimated by the the I-MaxEnt model, while the high susceptibility zone was not well predicated. Although the ROC curves and field validation all indicate the higher accuracy of the I-LR model, the presence of contiguous susceptible areas predicated by which may pose challenges to identify and mitigate collapse, landslides disaster in practice. Future studies should be carried out, such as the adjustment of sampling ratio or modification on proposed models to reduce the misclassification of landslide susceptibility zone.

## 5. Conclusions

Landslide is a major geological disaster, and timely and accurate delineation of landslide susceptibility area is of great significance for disaster defence and policy formulation. In this study, the information value-maximum entropy coupling model (I-MaxEnt) and the information value-logistic regression coupling model (I-LR) are proposed to assess landslide susceptibility in the economic zone of the northern slopes of the Tianshan Mountains, combining the traditional statistical methods and the machine learning model, after a detailed evaluation of the information value model (I), the logistic regression model (LR) and the maximum entropy model (MaxEnt).The following conclusions were mainly obtained:

(1) Both the I-LR and I-MaxEnt models can provide an reliable results in the landslide. susceptibility assessment with the ROC-AUC values of 0.941 and 0.907, suggesting that the I-LR model has a superior predictive accuracy;
(2) According to the results of two coupling models, the importance of three factors, namely distance from the water system, engineering geological rock formation, and distance from the road, is relatively high, which is reasonable. Based on the actual situation in the research area, the slope factor also plays a crucial role in the occurrence of disasters. Compared with the results of the two models, the I-LR evaluation model is more reasonable.And the landslide with a high susceptibility level in Miaoergou was taken as an example for field validation, the comparative analysis of which indicated the higher prediction accuracy of the I-LR model;
(3) The very highly susceptible zone are primarily linearly distributed in these regions with intense human activities along roads and river. Whereas, these high and moderate susceptibility zones are generally located in the foothills and low hilly.The implement of engineering stabilization measures and timely detection of hidden dangers in these areas are highly recommended.
